# Supplementary material for: High-molecular weight DNA extraction, clean-up and size selection for long-read sequencing
Source: PLoS One. 2021 Jul 15;16(7):e0253830. doi: 10.1371/journal.pone.0253830 (PMC8282028; doi:10.1371/journal.pone.0253830)
Supplement: S1 Protocol collection — (PDF) [file pone.0253830.s001.pdf]

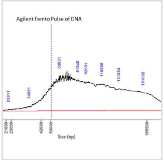

Feb 26, 2021

High-molecular weight DNA extraction, clean-up and size selection for long-read sequencing

Ashley Jones[1], Cynthia Torkel[1], David Stanley[1], Jamila Nasim[1], Justin Borevitz[1], Benjamin Schwessinger[1]

[1]Research School of Biology, Australian National University, Canberra, ACT, Australia

1 Works for me dx.doi.org/10.17504/protocols.io.bss7nehn

High molecular weight DNA extraction from all kingdoms
Tech. support email: **See@each.protocol**

Ashley Jones
Australian National University

SUBMIT TO PLOS ONE

ABSTRACT

Rapid advancements in long-read sequencing technologies have transformed sequencing read lengths from bps to Mbps, which has enabled chromosome-scale genome assemblies. However, read lengths are now becoming limited by the extraction of pure high-molecular weight DNA suitable for long-read sequencing, which is particularly challenging in plants and fungi. To overcome this, we present a protocol collection; high-molecular weight DNA extraction, clean-up and size selection for long-read sequencing. We optimised a gentle magnetic bead based high-molecular weight DNA extraction, which is presented here in detail. The protocol circumvents spin columns and high-centrifugation, to limit DNA fragmentation. The protocol is scalable based on tissue input, which can be used on many species of plants, fungi, reptiles, insects and bacteria. It is also cost effective compared to kit-based protocols and hence applicable at scale at low resource settings. An optional sorbitol wash is listed and is highly recommended for plant and fungal tissues. To further remove any remaining contaminants such as phenols and polysaccharides, optional DNA clean-up and size selection strategies are given. This protocol collection is suitable for all common long-read sequencing platforms, such as technologies offered by PacBio and Nanopore. Using these protocols, sequencing on the Oxford Nanopore MinION can achieve read length N50 values of 30-50 kb, with reads exceeding 200 kb and outputs ranging from 15-30 Gbp. This has been routinely achieved with eucalypts, acacias, rice, themeda, wheat, wheat rusts, various other fungi, geckos, skinks, ticks, ladybird beetles, caterpillars and E. coli.

DOI

dx.doi.org/10.17504/protocols.io.bss7nehn

COLLECTION CITATION

Ashley Jones, Cynthia Torkel, David Stanley, Jamila Nasim, Justin Borevitz, Benjamin Schwessinger 2021. High-molecular weight DNA extraction, clean-up and size selection for long-read sequencing. **protocols.io**
https://dx.doi.org/10.17504/protocols.io.bss7nehn

LICENSE

This is an open access collection distributed under the terms of the Creative Commons Attribution License, which permits unrestricted use, distribution, and reproduction in any medium, provided the original author and source are credited

CREATED

Feb 26, 2021

LAST MODIFIED

Feb 26, 2021

Citation: Ashley Jones, Cynthia Torkel, David Stanley, Jamila Nasim, Justin Borevitz, Benjamin Schwessinger (02/26/2021). High-molecular weight DNA extraction, clean-up and size selection for long-read sequencing. https://dx.doi.org/10.17504/protocols.io.bss7nehn

This is an open access protocol distributed under the terms of the **Creative Commons Attribution License** (https://creativecommons.org/licenses/by/4.0/), which permits unrestricted use, distribution, and reproduction in any medium, provided the original author and source are credited

COLLECTION INTEGER ID

47679

ABSTRACT

Rapid advancements in long-read sequencing technologies have transformed sequencing read lengths from bps to Mbps, which has enabled chromosome-scale genome assemblies. However, read lengths are now becoming limited by the extraction of pure high-molecular weight DNA suitable for long-read sequencing, which is particularly challenging in plants and fungi. To overcome this, we present a protocol collection; high-molecular weight DNA extraction, clean-up and size selection for long-read sequencing. We optimised a gentle magnetic bead based high-molecular weight DNA extraction, which is presented here in detail. The protocol circumvents spin columns and high-centrifugation, to limit DNA fragmentation. The protocol is scalable based on tissue input, which can be used on many species of plants, fungi, reptiles, insects and bacteria. It is also cost effective compared to kit-based protocols and hence applicable at scale at low resource settings. An optional sorbitol wash is listed and is highly recommended for plant and fungal tissues. To further remove any remaining contaminants such as phenols and polysaccharides, optional DNA clean-up and size selection strategies are given. This protocol collection is suitable for all common long-read sequencing platforms, such as technologies offered by PacBio and Nanopore. Using these protocols, sequencing on the Oxford Nanopore MinION can achieve read length N50 values of 30-50 kb, with reads exceeding 200 kb and outputs ranging from 15-30 Gbp. This has been routinely achieved with eucalypts, acacias, rice, themeda, wheat, wheat rusts, various other fungi, geckos, skinks, ticks, ladybird beetles, caterpillars and E. coli.

FILES

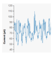

Scalable high-molecular weight DNA extraction for long-read sequencing

Version 1

by Ashley Jones, Australian National University

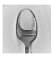

Sorbitol washing complex homogenate for improved DNA extractions

Version 1

by Ashley Jones, Australian National University

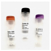

DNA clean-up and size selection for long-read sequencing

Version 3

by Ashley Jones, Australian National University

Citation: Ashley Jones, Cynthia Torkel, David Stanley, Jamila Nasim, Justin Borevitz, Benjamin Schwessinger (02/26/2021). High-molecular weight DNA extraction, clean-up and size selection for long-read sequencing. https://dx.doi.org/10.17504/protocols.io.bss7nehn

This is an open access protocol distributed under the terms of the **Creative Commons Attribution License** (https://creativecommons.org/licenses/by/4.0/), which permits unrestricted use, distribution, and reproduction in any medium, provided the original author and source are credited

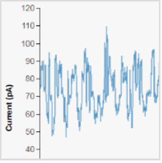

Oct 19, 2020

Scalable high-molecular weight DNA extraction for long-read sequencing

In 1 collection

Ashley Jones[1], Cynthia Torkel[1], David Stanley[1], Jamila Nasim[1], Justin Borevitz[1], Benjamin Schwessinger[1]

[1]Research School of Biology, Australian National University, Canberra, ACT, Australia

1 Works for me dx.doi.org/10.17504/protocols.io.bnjhmcj6

High molecular weight DNA extraction from all kingdoms
Tech. support email: **See@each.protocol**

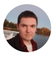

Ashley Jones
Australian National University

SUBMIT TO PLOS ONE

ABSTRACT

With rapid advances in long-read DNA sequencing technologies, it is becoming possible to resolve complex genomes, including repetitive, polyploid plant genomes. Despite the technology being available, a challenge persists: the extraction of pure high molecular weight DNA suitable for long-read sequencing. This is particularly true of native plants, crops and fungi. To resolve this, we optimised a gentle magnetic bead based high-molecular weight DNA extraction free of columns and high-centrifugation, to limit DNA fragmentation. A protocol that is scalable based on tissue input is presented, that can be used on many species of plants, fungi, reptiles, insects and bacteria. An optional sorbitol wash is listed and is highly recommended for plant tissues. To remove any remaining contaminants such as phenols and polysaccharides, two optional DNA clean-up and size selection strategies are given. Sequencing with Oxford Nanopore Technologies MinION, we can approximately obtain over 15-30 Gbp of sequencing from a single MinION flow cell with N50 values 30-50 kb. This has been routinely achieved with eucalypts, acacias, rice, themeda, wheat, wheat rusts, various other fungi, geckos, skinks, ticks, ladybird beetles, caterpillars and *E. coli*.

DOI

dx.doi.org/10.17504/protocols.io.bnjhmcj6

PROTOCOL CITATION

Ashley Jones, Cynthia Torkel, David Stanley, Jamila Nasim, Justin Borevitz, Benjamin Schwessinger 2020. Scalable high-molecular weight DNA extraction for long-read sequencing. **protocols.io**
https://dx.doi.org/10.17504/protocols.io.bnjhmcj6

COLLECTIONS

High-molecular weight DNA extraction, clean-up and size selection for long-read sequencing

LICENSE

This is an open access protocol distributed under the terms of the Creative Commons Attribution License, which permits unrestricted use, distribution, and reproduction in any medium, provided the original author and source are credited

CREATED

Oct 19, 2020

LAST MODIFIED

Oct 19, 2020

Citation: Ashley Jones, Cynthia Torkel, David Stanley, Jamila Nasim, Justin Borevitz, Benjamin Schwessinger (10/19/2020). Scalable high-molecular weight DNA extraction for long-read sequencing. https://dx.doi.org/10.17504/protocols.io.bnjhmcj6

This is an open access protocol distributed under the terms of the **Creative Commons Attribution License** (https://creativecommons.org/licenses/by/4.0/), which permits unrestricted use, distribution, and reproduction in any medium, provided the original author and source are credited

PROTOCOL INTEGER ID

43337

PARENT PROTOCOLS

Part of collection

High-molecular weight DNA extraction, clean-up and size selection for long-read sequencing

GUIDELINES

This protocol is based on the following publication. When citing, please also note the original publication below.

Mayjonade, B., Gouzy, J., Donnadieu, C., Pouilly, N., Marande, W., Callot, C., Langlade, N., and Munos, S. (2016). Extraction of high-molecular-weight genomic DNA for long-read sequencing of single molecules. BioTechniques **61,** 203-205.

MATERIALS TEXT

Chemicals for lysis buffer

- Ethylene-bis(oxyethylenenitrilo)tetraacetic acid (EGTA) (optional; necessary for wheat)
- Ethylenediaminetetraacetic acid (EDTA)
- Polyvinylpyrrolidone 40 (PVP-40)
- Sodium chloride (NaCl)
- Sodium dodecyl sulfate (SDS)
- Sodium metabisulfite
- Tris(hydroxymethyl)aminomethane hydrochloride (TRIS-HCl)
- Water (Milli-Q)

Other solutions

- Ethanol (100% and 70%)
- Lysozyme (bacterial culture preps only)
- Nuclease-free water (UltraPure™ Thermo Fisher Scientific 10977015)
- 5 M Potassium acetate
- Proteinase K (20 mg/mL 2x1 mL NEB P8107S)
- RNAse A (PureLink 20 mg/mL 25 mL, Thermo Fisher Scientific 12091039)
- 10 mM TRIS-HCl pH 8

2% Sera-Mag beads solution

- Sera-Mag SpeedBead Carboxylate-Modified Magnetic Particles (Hydrophobic), 15 mL (GE Healthcare /Cytiva /Thermo Fisher Scientific product 65152105050250).
- First prepare buffer without the beads. Let the Sera-Mag beads come to room temperature.

Component	Target concentration	MW	Stock concentration	From stock (10 mL)	From stock (50 mL)
Polyethylene glycol (PEG) 8,000	18%	8,000	25%	7.2 mL	36 mL
NaCl	1 M	58.44	5 M	2 mL	10 mL
TRIS-HCl pH 8.0	10 mM	121.14	1 M	100 µL	500 µL
EDTA pH 8.0	1 mM	292.24	0.5 M	20 µL	100 µL
Tween 20	0.05%	1,227.54	10%	50 µL	250 µL

- Homogenise Sera-Mag beads thoroughly by shaking and swirling.
- Prepare 2% Sera-Mag beads (200 µL for 10 mL buffer) by washing 4 times with water to remove sodium azide. Magnetise, remove supernatant, add 1 mL H_2O, flick tube, repeat.

Citation: Ashley Jones, Cynthia Torkel, David Stanley, Jamila Nasim, Justin Borevitz, Benjamin Schwessinger (10/19/2020). Scalable high-molecular weight DNA extraction for long-read sequencing. https://dx.doi.org/10.17504/protocols.io.bnjhmcj6

This is an open access protocol distributed under the terms of the **Creative Commons Attribution License** (https://creativecommons.org/licenses/by/4.0/), which permits unrestricted use, distribution, and reproduction in any medium, provided the original author and source are credited

- Resuspend the clean Sera-Mag beads in 600 µL nuclease-free water. Transfer into the buffer prepared.
- Store at 4°C for up to 6 months.

Binding buffer solution

- Mix until the solution becomes clear. If PEG 8,000 is not dissolved, it can lead to a poor yield as PEG 8,000 makes DNA to bind to the beads.

Component	Target concentration	MW	Stock concentration	From stock (50 mL)	From stock (1L)
Polyethylene glycol (PEG) 8,000	20%	8,000	powder	10 g	200 g
NaCl	3 M	58.44	powder	8.75 g	175 g

Special equipment

- Liquid nitrogen
- Mortar and pestle (medium and large preps)
- Ball bearings and TissueLyser (Qiagen) or equivalent (small prep only)
- Magnetic rack (for 2 mL Eppendorf tubes and/or 15 mL and/or 50 mL Falcon Tubes)
- 2 mL Eppendorf tubes (small preps)
- 50 mL Falcon tubes (medium preps)
- 250 mL Schott bottle (large prep only)
- 1.5 mL DNA LoBind Eppendorf tubes (e.g. 022431021) (optional but optimal)
- 200 µL wide-bore pipette tips (e.g. Vertex 4290-00) (optional)
- Sieve or mesh with a funnel, 300 µm (e.g. Kartell disk membrane; ART 844) (large prep only)

PREPARATION

1 Set a water bath (or equivalent alternative) to 55°C (temperature range 50-60°C)

Will be used to dissolve lysis buffer and also used during lysis (30-90 min).

2 Prepare fresh lysis buffer based on the prep size and heat at 55°C in water bath until ready to use.

Prep size	Tissue input	Amount needed per sample
Small (2 mL tube prep)	100-200 mg	750 µL
Medium (50 mL Falcon tube prep)	1-3 g	20 mL
Large (250 mL Schott bottle prep)	≤ 30 g	100 mL

Citation: Ashley Jones, Cynthia Torkel, David Stanley, Jamila Nasim, Justin Borevitz, Benjamin Schwessinger (10/19/2020). Scalable high-molecular weight DNA extraction for long-read sequencing. https://dx.doi.org/10.17504/protocols.io.bnjhmcj6

This is an open access protocol distributed under the terms of the **Creative Commons Attribution License** (https://creativecommons.org/licenses/by/4.0/), which permits unrestricted use, distribution, and reproduction in any medium, provided the original author and source are credited

Reagent	Target concentration	MW	Stock	From stock (10 mL)
Polyvinylpyrrolidone 40 (PVP-40)	1%	40,000	10%	1 mL
Sodium metabisulfite	1%	190.11	10%	1 mL
Sodium chloride (NaCl)	0.5 M	58.44	5 M	1 mL
TRIS-HCl pH 8.0	100 mM	121.14	1 M	1 mL
EDTA pH 8.0	50 mM	292.24	0.5 M	1 mL
Sodium dodecyl sulfate (SDS)	2%	288.37	20%	1 mL
Water (Milli-Q)	NA	-	-	4 mL
EGTA pH 8 (optional, see below)	6 mM	380.35	0.5 M	120 µL

- *Prepare lysis buffer fresh on the day of use for optimal results.*
- *The solution should be clear before use.*
- *EGTA is recommended for wheat and very soft plant tissues that wilt fast and/or have high amounts of endogenous DNases.*
- *EDTA has a high-affinity for Mg^{2+}, EGTA has a high-affinity for Ca^{2+}.*
- *Prokaryotic DNases are dependent on Mg^{2+}, plant DNAses Ca^{2+}.*

3 Ensure there is enough 5 M potassium acetate, binding buffer and Sera-Mag beads to carry out the prep size and quantity (listed in Materials).

HOMOGENISATION

4 [Bacteria cultures only] Pellet cells in a 2 mL tube or 50 mL Falcon tube by centrifuging at 5,000 rcf for 10 min at room temperature and discard growth medium. Skip next step (grinding) and proceed to adding lysis buffer.

5 Grind tissue to a fine powder, keeping frozen with liquid nitrogen.

Prep size	Tissue input	Recommendation
Small (2 mL tube prep)	100-200 mg	1-3 ball bearings in 2 mL tube, place in TissueLyser (Qiagen) for 2 min at 25 Hz.
Medium (50 mL Falcon tube prep)	1-3 g	Mortar and pestle, transfer to a 50 mL Falcon tube.
Large (250 mL Schott bottle prep)	≤ 30 g	Mortar and pestle, or blender. Transfer to 250 mL Schott bottle.

SORBITOL WASH (OPTIONAL)

6 [Optional] If the sample is likely to have a high amount of sugars, oils and/or other endogenous chemicals present (e.g. plant tissue), perform a sorbitol wash. See the following protocol:

Citation: Ashley Jones, Cynthia Torkel, David Stanley, Jamila Nasim, Justin Borevitz, Benjamin Schwessinger (10/19/2020). Scalable high-molecular weight DNA extraction for long-read sequencing. https://dx.doi.org/10.17504/protocols.io.bnjhmcj6

This is an open access protocol distributed under the terms of the **Creative Commons Attribution License** (https://creativecommons.org/licenses/by/4.0/), which permits unrestricted use, distribution, and reproduction in any medium, provided the original author and source are credited

[Sorbitol washing complex homogenate for improved DNA extractions](#)

Jones, A., and Schwessinger, B. (2020). Sorbitol washing complex homogenate for improved DNA extractions. Protocols.io v1, dx.doi.org/10.17504/protocols.io.beuvjew17506.

CELL LYSIS

7 Add lysis buffer to tissue. Vortex or shake vigorously to mix.

Prep size	Lysis buffer to add
Small (2 mL tube prep)	750 µL
Medium (50 mL Falcon tube prep)	Bring volume to 20 mL
Large (250 mL Schott bottle prep)	Bring volume to 100 mL

Lysis buffer should be 1-2x the approximate volume of the ground tissue.

8 [Bacteria cultures only] Add a small scoop of lysozyme to the lysis reaction, approximately 2-4 mg/mL.

9 Add RNAse A and Proteinase K to the solution. Mix by swirling and inverting.

[Bacteria cultures only] Do not add Proteinase K yet.

Prep size	Lysis volume	RNAse A (20 mg/mL stock)	Proteinase K (20 mg/mL stock)
Small (2 mL tube prep)	750 µL	8 µL (213 µg/mL final)	4 µL (107 µg/mL final)
Medium (Falcon tube prep)	20 mL	200 µL (200 µg/mL final)	100 µL (100 µg/mL final)
Large (Schott bottle prep)	100 mL	400 µL (80 µg/mL final)	200 µL (40 µg/mL final)

Proteinase K and RNAse A can co-exist in the same solution, even in the presence of EDTA.
- *RNAse A is highly resistant to proteolysis by Proteinase K.*
- *Activity of RNAse A and Proteinase K is not dependent on ion cofactors, EDTA has no effect.*
- *Both are recommended to be 50-100 µg/mL.*

Citation: Ashley Jones, Cynthia Torkel, David Stanley, Jamila Nasim, Justin Borevitz, Benjamin Schwessinger (10/19/2020). Scalable high-molecular weight DNA extraction for long-read sequencing. https://dx.doi.org/10.17504/protocols.io.bnjhmcj6

This is an open access protocol distributed under the terms of the **Creative Commons Attribution License** (https://creativecommons.org/licenses/by/4.0/), which permits unrestricted use, distribution, and reproduction in any medium, provided the original author and source are credited

- *Qiagen DNeasy plant kits use as much as 2,000 µg/mL RNAse A.*
- *RNAse T has less DNase activity (still present), but was less effective at degrading RNA.*
- *RNAse A is active at temperatures 15-70°C (optimal at 60°C), pH 6-10 (optimal pH 7.6).*
- *Proteinase K is active at temperatures 20-60°C (optimal 50-60°C), pH 4-12 (optimal pH 8).*
- *Proteinase K degrades proteins in the presence of detergents, including SDS.*
- *Proteinase K activity is stimulated if up to 2% SDS or 4 M urea is present in the reaction.*
- *Lysozyme activity varies based on where it was purified from. Generally, active at temperatures 20-55°C (optimal at 30-50°C), pH 6-9 (optimal pH 6.2).*

10 Incubate the samples at 55°C, shaking at ~450 rpm if possible. Recommended time:

Prep size	Incubation time
Small (2 mL tube prep)	60 min
Medium (50 mL Falcon tube prep)	60 min
Large (250 mL Schott bottle prep)	90-120 min
[Bacteria cultures only]	30 min

Higher temperatures and longer incubations lead to DNA damage.

11 [Bacteria cultures only] Add Proteinase K (see previous table) and incubate at 55°C for an additional 30 min.

CONTAMINANT REMOVAL

12 [Large prep only] Filter the homogenate using a sieve (or disk membrane mesh placed in a funnel) into a new 250 mL Schott bottle. Forcibly squeeze out as much residual homogenate from the debris as possible. Then split the homogenate into 50 mL Falcon tubes, bringing the volume to 20 mL for each.

13 Add 1/3 volume of 5 M Potassium Acetate and mix by inverting to precipitate the proteins and the polysaccharides that will complex with SDS.

Prep size	Potassium acetate
Small (2 mL tube prep)	~250 µL
Medium (50 mL Falcon tube prep)	~7 mL
Large (250 mL Schott bottle prep)	~7 mL per 50 mL Falcon tube

14 Incubate on ice (4°C) for 10 min (don't rotate, DNA vulnerable).

15 Centrifuge at 5,000 rcf for 5 min at 4°C.

Citation: Ashley Jones, Cynthia Torkel, David Stanley, Jamila Nasim, Justin Borevitz, Benjamin Schwessinger (10/19/2020). Scalable high-molecular weight DNA extraction for long-read sequencing. https://dx.doi.org/10.17504/protocols.io.bnjhmcj6

This is an open access protocol distributed under the terms of the **Creative Commons Attribution License** (https://creativecommons.org/licenses/by/4.0/), which permits unrestricted use, distribution, and reproduction in any medium, provided the original author and source are credited

16 Transfer supernatant to a new tube, centrifuge again at 5,000 rcf for 10 min at 4°C.

17 Transfer supernatant to a new tube.

DNA BINDING AND WASH

18 Add approximately an equal volume of binding buffer or more. Utilise the capacity of the tube, leaving only a small space for adding Sera-Mag beads. Approximate amounts:

Prep size	Sample volume	Binding buffer
Small (2 mL tube prep)	~950 µL	Bring volume to 1.90 mL
Medium (50 mL Falcon tube prep)	~25 mL	Bring volume to 50 mL
Large (250 mL Schott bottle prep)	~25 mL per 50 mL Falcon tube	Bring volume to 50 mL

Adding more buffer can increase to yield, especially if the sample has a lot of contaminants. More shorter fragments will also be recovered, but can be size selected against later.

19 Thoroughly mix the 2% Sera-Mag beads and add them to the sample:

Prep size	Sera-Mag beads
Small (2 mL tube prep)	100 µL
Medium (50 mL Falcon tube prep)	1 mL
Large (250 mL Schott bottle prep)	1 mL per Falcon or 10 mL overall

20 Mix by inverting the tube 20 times. Incubate with gentle mixing using a rotator or a shaker platform at room temperature.

Citation: Ashley Jones, Cynthia Torkel, David Stanley, Jamila Nasim, Justin Borevitz, Benjamin Schwessinger (10/19/2020). Scalable high-molecular weight DNA extraction for long-read sequencing. https://dx.doi.org/10.17504/protocols.io.bnjhmcj6

This is an open access protocol distributed under the terms of the **Creative Commons Attribution License** (https://creativecommons.org/licenses/by/4.0/), which permits unrestricted use, distribution, and reproduction in any medium, provided the original author and source are credited

Prep size	Recommended time
Small (2 mL tube prep)	≥ 10 min
Medium (50 mL Falcon tube prep)	≥ 60 min or overnight
Large (250 mL Schott bottle prep)	≥ 120 min or overnight

21 Place the tube in a magnetic rack until the solution becomes clear.

Prep size	Recommended time
Small (2 mL tube prep)	≥ 10 min
Medium (50 mL Falcon tube prep)	≥ 30 min
Large (250 mL Schott bottle prep)	≥ 120 min or overnight

22 Remove the supernatant without disturbing the beads, keeping the tube on the magnetic rack.

Small preps can be done by pipetting, larger preps by decanting.

23 Wash the beads by filling the tube with 70% ethanol, let beads settle if disturbed, and decant out the ethanol.

24 Repeat the ethanol wash another 2 times, or until satisfied the beads are clean.

If the beads are very dirty, remove the tube from the magnetic rack, resuspend beads by flicking the tube, magnetise until clear, remove supernatant, repeat washing.

25 For medium and large preps, transfer the beads into a 2 mL Eppendorf tube. This can be done by taking the Falcon tube off the rack, adding 1 mL of 70% ethanol to dislodge the beads, transfer suspension to an Eppendorf tube. Repeat with another 1 mL to ensure all beads are transferred. Place the 2 mL Eppendorf tube on the magnetic rack, remove supernatant and repeat the process with other Falcon tubes until all beads are in the same eppendorf tube.

Do not let the beads dry during the process, keep the beads wet with ethanol and perform the process quickly. Larger preps can be done at the sink to discard ethanol easily.

26 Remove all traces of ethanol and let the beads air dry for 1-4 min.

Important! *Do not let the beads dry completely, they will crack and significantly reduce DNA recovery.*

Citation: Ashley Jones, Cynthia Torkel, David Stanley, Jamila Nasim, Justin Borevitz, Benjamin Schwessinger (10/19/2020). Scalable high-molecular weight DNA extraction for long-read sequencing. https://dx.doi.org/10.17504/protocols.io.bnjhmcj6

This is an open access protocol distributed under the terms of the **Creative Commons Attribution License** (https://creativecommons.org/licenses/by/4.0/), which permits unrestricted use, distribution, and reproduction in any medium, provided the original author and source are credited

27 Remove tube from magnetic rack and add 10 mM TRIS-HCl pH 8 (or nuclease-free water) to the beads, gently resuspending (use wide-bore pipette tip if available). Gently tapping the tube is also suitable.

If the solution is very thick and cloudy white, there is a large yield; consider slowly increasing the elution volume (in some cases, double or triple the volume, e.g. large genomes, high cell density).

Prep size	Recommended elution
Small (2 mL tube prep)	50 µL
Medium (50 mL Falcon tube prep)	200 µL
Large (250 mL Schott bottle prep)	500 µL

Larger volume is based on size selection protocols, e.g. PippinHT input across x1 whole cassette or a DNA clean-up protocol.

28 Incubate at room temperature for at least 10 min.

29 Place the tubes in the magnetic rack until the solution becomes clear.

Highly concentrated DNA will take a long time. The tube can be left on the magnetic rack overnight in the fridge, or increase the elution volume.

30 Transfer the supernatant (contains DNA) to a 1.5 mL Eppendorf tube, avoiding any carry-over of beads. Use DNA LoBind tube if available.

31 Perform an additional, second elution on the beads (same volume as previous), remove from magnet, resuspend, incubate, magnetise and transfer to another 1.5 mL Eppendorf tube. Use DNA LoBind tube if available.

Save this second elution for other applications such as running a pulse field gel.

32 Quantify the DNA on a Nanodrop and a Qubit fluorometer (dsDNA broad-range assay) (both instruments from Thermo Fisher Scientific). The DNA is still crude and will likely have other contaminants present.

- *For Nanodrop, use 1 µL. As the DNA is crude, it may give a large over-estimation of concentration.*
- *Qubit fluorometer is the most reliable. However, it is highly dependent on the accuracy of the amount pipetted. Use 2 µL when sample is plentiful. Using 1 µL is prone to pipetting errors.*
- *For pure DNA, Nanodrop:Qubit is 1:1, 260/280 is 1.8 and 260/230 is 2.0.*
- *DNA yield can be 20-500 µg.*

33 Store DNA at 4°C to prevent cycles of freeze-thawing that shear the DNA.

Citation: Ashley Jones, Cynthia Torkel, David Stanley, Jamila Nasim, Justin Borevitz, Benjamin Schwessinger (10/19/2020). Scalable high-molecular weight DNA extraction for long-read sequencing. https://dx.doi.org/10.17504/protocols.io.bnjhmcj6

This is an open access protocol distributed under the terms of the **Creative Commons Attribution License** (https://creativecommons.org/licenses/by/4.0/), which permits unrestricted use, distribution, and reproduction in any medium, provided the original author and source are credited

No effects on DNA integrity have been noticed for samples stored at 4°C for 12 months.

34 For some species, the DNA may be pure enough to proceed to sequencing. If the DNA is crude (likely for plant samples), consider DNA clean-up and size slection options below.

DNA CLEAN-UP AND SIZE SELECTION OPTION 1: SHORT-READ ELIMINATOR

35 If the DNA is clear, has small quantities of impurities, consider utilising a Short-Read Elimator kit from Circulomics.

If the DNA is discoloured with visbible impurities, it is best for perform a DNA clean-up first before using the Short-Read Elimator kit. See the following protocol:

DNA clean-up and size selection for long-read sequencing

Jones, A., Purushotham, N., Nasim, J., and Schwessinger, B. (2020). DNA clean-up and size selection for long-read sequencing. Protocols.io v3, dx.doi.org/10.17504/protocols.io.betdjei17506.

It has been observed that the Short-Read Eliminator kit can result in and unexpected increased absorbance/fluorescence from Nanodrop/Qubit results. If this is seen, consider using more DNA input into the sequencing library prep than recommended. Perhaps the DNA double helix becomes altered or unwound. Alternatively, what is traditionally measured as DNA concentration has always been an under-estimate.

DNA CLEAN-UP AND SIZE SELECTION OPTION 2: GEL PURIFICATION BY PIPPIN PREP

36 For most recalcitrant plants, the DNA will still have a high quantity of impurities and DNA fragmentation is inevitable. Gel purification is an ideal solution to both problems. Proceed to gel purification by Pippin Prep (Sage Science) or equivalent.

37 If a precipitant is noticebale in the DNA solution, briefly spin down and take the upper aqueous layer to gel purification below.

For some samples, this may be carbohydrates, which will be removed during gel purification. DNA is in the aqueous layer. It is possible 4°C promotes this precipitation.

38 Using a PippinHT (Sage Science) or similar automated electrophoresis product, gel purify approximately 30 µg, following the manufacturer's instructions. A 20 kb high pass separation is recommended, however if DNA is limited, 15 kb high pass is suitable. The PippinHT has 12 lanes, however a lane pair needs be dedicated to an external ladder, leaving 10 lanes for samples. 20 µL of DNA goes into each lane (therefore 200 µL elution in previous section). The manufacturer recommends a maximum 1.5 µg per lane (15 µg total per cassette), however, can be safely overloaded to 3 µg per lane (30 µg total) without noticable consequences. Overloading further slows the migration of longer fragments and shorter fragments are eluted as the size selection will not be precise.

If the sample if very dirty and may not electrophorese correctly, consider a chloroform: isoamyl alcohol 24:1 clean-up before using the PippinHT.

39 After separation, wait at least 45 min (hours or overnight is suitable), to aid elution and recovery.

Citation: Ashley Jones, Cynthia Torkel, David Stanley, Jamila Nasim, Justin Borevitz, Benjamin Schwessinger (10/19/2020). Scalable high-molecular weight DNA extraction for long-read sequencing. https://dx.doi.org/10.17504/protocols.io.bnjhmcj6

This is an open access protocol distributed under the terms of the **Creative Commons Attribution License** (https://creativecommons.org/licenses/by/4.0/), which permits unrestricted use, distribution, and reproduction in any medium, provided the original author and source are credited

40 Collect the contents of all elution wells into a 1.5 mL Eppendorf tube (approx. 300 µL). Use DNA LoBind tube if available.

41 Add 30 µL of 0.1% tween in electrophoresis buffer to each elution well (provided in kit). Wait for 5 min and then transfer the contents to the same 1.5 mL Eppendorf tube (another 300 µL, tube total is approx. 600 µL).

42 Add 1.2x binding buffer (approx. 720 µL), and 100 µL of 2% Sera-Mag beads to the 1.5 mL Eppendorf tube. Incubate for 5-10 min at room temperature.

43 Place on a magnetic rack for 5 min, or until the solution becomes clear. Discard the supernatant.

44 Keeping the tube on the magnetic rack, add 1 mL of freshly prepared 70% ethanol. Discard the ethanol and repeat for a second ethanol wash.

45 Remove all traces of ethanol and let the beads air dry for 1-4 min.

> ***Important!*** *Do not let the beads dry completely, they will crack and significantly reduce DNA recovery.*

46 Remove the tube from the magnetic rack and elute with 50 µL of nuclease-free water. Incubate for 10 min at room temperature.

> *A maximum DNA volume of 48 µL is used in an Oxford Nanopore ligation prep (e.g. SQK-LSK109).*

47 Place on a magnetic rack for 5 min, or until the solution becomes clear. Transfer eluted DNA to a new 1.5 mL Eppendorf tube. Use DNA LoBind tube if available. Use a wide-bore pipette tip if available.

48 Perform an additional, second elution on the beads (same volume as previous), remove from magnet, resuspend, incubate, magnetise and transfer to another 1.5 mL Eppendorf tube. Use DNA LoBind tube if available.

> *Save this second elution for other applications such as running a pulse field gel.*

49 Quantify the DNA on a Nanodrop and a Qubit fluorometer (dsDNA broad-range assay) (both instruments from Thermo Fisher Scientific). The DNA should be pure, free of contaminants.

- *For Nanodrop, use 1 µL.*

Citation: Ashley Jones, Cynthia Torkel, David Stanley, Jamila Nasim, Justin Borevitz, Benjamin Schwessinger (10/19/2020). Scalable high-molecular weight DNA extraction for long-read sequencing. https://dx.doi.org/10.17504/protocols.io.bnjhmcj6

This is an open access protocol distributed under the terms of the **Creative Commons Attribution License** (https://creativecommons.org/licenses/by/4.0/), which permits unrestricted use, distribution, and reproduction in any medium, provided the original author and source are credited

- *Qubit fluorometer is highly dependent on the accuracy of the amount pipetted. Use 2 µL when sample is plentiful. Using 1 µL is prone to pipetting errors.*
- *For pure DNA, Nanodrop:Qubit is 1:1, 260/280 is 1.8 and 260/230 is 2.0.*
- *Expect 20-30% recovery relative to total input (~6-9 µg out of 30 µg).*

50 Store DNA at 4°C to prevent cycles of freeze-thawing that shear the DNA.

No effects on DNA integrity have been noticed for samples stored at 4°C for 12 months.

Citation: Ashley Jones, Cynthia Torkel, David Stanley, Jamila Nasim, Justin Borevitz, Benjamin Schwessinger (10/19/2020). Scalable high-molecular weight DNA extraction for long-read sequencing. https://dx.doi.org/10.17504/protocols.io.bnjhmcj6

This is an open access protocol distributed under the terms of the **Creative Commons Attribution License** (https://creativecommons.org/licenses/by/4.0/), which permits unrestricted use, distribution, and reproduction in any medium, provided the original author and source are credited

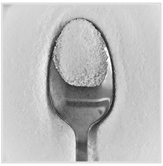

May 20, 2020

Sorbitol washing complex homogenate for improved DNA extractions

In 1 collection

Ashley Jones[1], Benjamin Schwessinger[1]

[1]Research School of Biology, Australian National University, Canberra, ACT, Australia

2 Works for me

dx.doi.org/10.17504/protocols.io.beuvjew6

High molecular weight DNA extraction from all kingdoms
Tech. support email: **See@each.protocol**

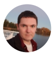

Ashley Jones
Australian National University

SUBMIT TO PLOS ONE

ABSTRACT

The extraction of pure DNA can be challenging due to the presence of sugars, oils and other endogenous chemicals present within biological samples. This is particularly true for many plants and fungi due to the presence of secondary metabolites such as polyphenols and polysaccharides. Polyphenols within the cytosol can become irreversibly DNA-bound after cell lysis and polysaccharides can co-precipitate with DNA during the extraction. Sorbitol is an osmotically active sugar alcohol and washing homogenate with sorbitol before cell lysis has been shown to significantly improve the purity of DNA extractions. Sorbitol does not pass cell membranes and likely acts by drawing the cytosol out of the cell. Therefore polyphenols and polyscacharides would be removed.

DOI

dx.doi.org/10.17504/protocols.io.beuvjew6

PROTOCOL CITATION

Ashley Jones, Benjamin Schwessinger 2020. Sorbitol washing complex homogenate for improved DNA extractions. **protocols.io**
https://dx.doi.org/10.17504/protocols.io.beuvjew6

COLLECTIONS

High-molecular weight DNA extraction, clean-up and size selection for long-read sequencing

LICENSE

This is an open access protocol distributed under the terms of the Creative Commons Attribution License, which permits unrestricted use, distribution, and reproduction in any medium, provided the original author and source are credited

CREATED

Apr 09, 2020

LAST MODIFIED

May 20, 2020

PROTOCOL INTEGER ID

35445

Citation: Ashley Jones, Benjamin Schwessinger (05/20/2020). Sorbitol washing complex homogenate for improved DNA extractions. https://dx.doi.org/10.17504/protocols.io.beuvjew6

This is an open access protocol distributed under the terms of the **Creative Commons Attribution License** (https://creativecommons.org/licenses/by/4.0/), which permits unrestricted use, distribution, and reproduction in any medium, provided the original author and source are credited

PARENT PROTOCOLS

Part of collection

High-molecular weight DNA extraction, clean-up and size selection for long-read sequencing

GUIDELINES

This protocol is based on the following publication. When citing, please also note the original publication below.

Inglis, P., Pappas, M.d.C., Resende, L. and Grattapaglia, D. (2018). Fast and inexpensive protocols for consistent extraction of high quality DNA and RNA from challenging plant and fungal samples for high-throughput SNP genotyping and sequencing applications. PLOS ONE **13**, 1-14.

MATERIALS TEXT

Dithiothreitol (DTT) or β-Mercaptoethanol
EDTA pH 8
Milli-Q water (MQW)
PVP 40,000
D-Sorbitol
Tris-HCl pH 8

PREPARATION

1 Prepare sorbitol wash solution. Approximately 2x volume of tube capacity will be used per sample. Sample tissue should not exceed 33% of tube capacity.

To prepare a 500 mL solution:

Reagent	Target concentration	Molecular weight	Stock concentration	Add from stock
D-Sorbitol	0.35 M	182.17	powder	31.88 g
PVP 40,000	1% (w/v)	40,000	powder	5 g
Tris-HCl pH 8	100 mM	157.60	1 M	50 mL
EDTA pH 8	5 mM	292.24	0.5 M	5 mL
MQW	NA	NA	NA	Bring to 500 mL

- *Store at 4°C for up to 6 months.*
- *DTT or β-Mercaptoethanol will be added freshly when used (below).*

HOMOGENISATION

2 Grind tissue to a fine powder, keep frozen with liquid nitrogen.

3 If homogenate is not in a tube (i.e. mortar and pestle), transfer to an appropriate sized tube.

Homogenate should not exceed 33% of tube capacity.

Citation: Ashley Jones, Benjamin Schwessinger (05/20/2020). Sorbitol washing complex homogenate for improved DNA extractions. https://dx.doi.org/10.17504/protocols.io.beuvjew6

This is an open access protocol distributed under the terms of the **Creative Commons Attribution License** (https://creativecommons.org/licenses/by/4.0/), which permits unrestricted use, distribution, and reproduction in any medium, provided the original author and source are credited

SORBITOL WASH

4 Fill the tube capacity with an excess of sorbitol wash solution.

5 Add DTT, approximate final concentration of 1 mM. Add 0.15 mg DTT per 1 mL of sorbitol wash. A tiny scoop of powder is sufficient. DTT MW = 154.253.

Alternatively add 1% β-Mercaptoethanol (v/v). Add 10 µL of β-Mercaptoethanol per 1 mL of sorbitol wash.

6 Shake, invert and vortex to mix thoroughly.

Ensure tissue is in suspension.

7 Centrifuge at 5,000 rcf for 5 min at room temperature.

Limited based on tubes rupturing if they contain ball bearings. Can also be reduced to 2,500 rcf.

8 Carefully decant the supernatant. Remove as much of the wash solution as possible without losing the pellet.

Supernatant should appear slightly cloudy or discoloured light yellow or brown.

9 If the supernatant was turbid, viscous or had dark discolouration, repeat the sorbitol wash.

DNA EXTRACTION

10 Proceed to a DNA extraction method of choice by adding lysis buffer to the pellet. Continue protocol as normal.

Citation: Ashley Jones, Benjamin Schwessinger (05/20/2020). Sorbitol washing complex homogenate for improved DNA extractions. https://dx.doi.org/10.17504/protocols.io.beuvjew6

This is an open access protocol distributed under the terms of the **Creative Commons Attribution License** (https://creativecommons.org/licenses/by/4.0/), which permits unrestricted use, distribution, and reproduction in any medium, provided the original author and source are credited

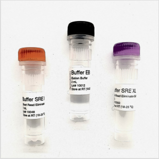

Version 3

Apr 09, 2020

DNA clean-up and size selection for long-read sequencing V.3

In 1 collection

Ashley Jones[1], Neeraj Purushotham[2], Jamila Nasim[1], Benjamin Schwessinger[1]

[1]Research School of Biology, Australian National University, Canberra, ACT, Australia;
[2]Institute for Agriculture and the Environment, Research and Innovation Division, University of Southern Queensland, Toowoomba, QLD, Australia

1 Works for me   dx.doi.org/10.17504/protocols.io.betdjei6

High molecular weight DNA extraction from all kingdoms
Tech. support email: **See@each.protocol**

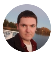

Ashley Jones
Australian National University

SUBMIT TO PLOS ONE

ABSTRACT

DNA extractions often contain impurities which limit the output of long-read sequencing technologies. Here a protocol is provided which removes impurities and size selects for longer fragments. To remove residual RNA and protein, an additional RNAse A and Proteinase K treatment is performed. A clean-up with chloroform: isoamyl alcohol (24:1) removes these proteins and other hydrophobic organics such as lipids. A low volume ethanol precipitation and wash is used to concentrate the DNA, hopefully also reducing polysaccharides. An optional needle shearing is described which can help create a more uniform DNA length to maximise sequencing output. Finally, a Short-Read Eliminator (SRE) kit by Circulomics is utilised for size selection, which also appears to clean the DNA. This was trialled for the sorghum rot fungus *Macrophomina phaseolina*, providing highly promising results with an Oxford Nanopore MinION. One strain yielded 13.71 Gbases with an N50 of 21.75 kb, another strain yielded 9.72 Gbases with an N50 of 43.50 kb. Similar results have been obtained with other fungi, plants, reptiles and insects.

DOI

dx.doi.org/10.17504/protocols.io.betdjei6

PROTOCOL CITATION

Ashley Jones, Neeraj Purushotham, Jamila Nasim, Benjamin Schwessinger 2020. DNA clean-up and size selection for long-read sequencing. **protocols.io**
https://dx.doi.org/10.17504/protocols.io.betdjei6

COLLECTIONS

High-molecular weight DNA extraction, clean-up and size selection for long-read sequencing

LICENSE

This is an open access protocol distributed under the terms of the Creative Commons Attribution License, which permits unrestricted use, distribution, and reproduction in any medium, provided the original author and source are credited

CREATED

Apr 08, 2020

LAST MODIFIED

Apr 09, 2020

Citation: Ashley Jones, Neeraj Purushotham, Jamila Nasim, Benjamin Schwessinger (04/09/2020). DNA clean-up and size selection for long-read sequencing. https://dx.doi.org/10.17504/protocols.io.betdjei6

This is an open access protocol distributed under the terms of the **Creative Commons Attribution License** (https://creativecommons.org/licenses/by/4.0/), which permits unrestricted use, distribution, and reproduction in any medium, provided the original author and source are credited

PROTOCOL INTEGER ID

35397

PARENT PROTOCOLS

Part of collection

High-molecular weight DNA extraction, clean-up and size selection for long-read sequencing

GUIDELINES

This protocol is optimsed for high-molecular weight DNA. For best results, avoid column based DNA extractions (which shear the DNA) and avoid using phenol (interferes with sequencing, can irreversibly bind to DNA).

MATERIALS TEXT

Chloroform: isoamyl alcohol (24:1 v/v)
1.5 mL Eppendorf DNA LoBind tubes
Ethanol (100% and 70%)
Needle (29 or 26 gauge) with syringe (optional shearing)
Proteinase K (20 mg/mL)
RNAse A (20 mg/mL)
Short Read Eliminator kit (Circulomics)
3 M Sodium acetate pH 5.2
10 mM Tris-HCl pH 8 (nuclease-free)
Water (nuclease-free)

RNA AND PROTEIN REMOVAL

1 Aliquot 10-30 µg of DNA into a 1.5 mL eppendorf tube. Increase volume to 200 µL with 10 mM Tris-HCl pH 8.

- *DNA quantification must be based on Qubit Fluorometer (Thermo Fisher Scientific), or similar device. A high quantity of RNA may be present.*
- *Volume can exceed 200 µL, maximum of 600 µL due to tube capacity at later steps (ethanol precipitation).*

2 Add RNAse A and Proteinase K

Enzyme	Stock	Quantity
100 µg/mL RNAse A	20 mg/mL	1 µL
100 µg/mL Proteinase K	20 mg/mL	1 µL

3 Incubate the samples at 50-60°C for 20 min, shaking at 400-900 rpm if possible.

CHLOROFORM CLEAN

4 Increase the volume to 600 µL with 10 mM Tris-HCl pH 8 (add 400 µL).

Citation: Ashley Jones, Neeraj Purushotham, Jamila Nasim, Benjamin Schwessinger (04/09/2020). DNA clean-up and size selection for long-read sequencing. https://dx.doi.org/10.17504/protocols.io.betdjei6

This is an open access protocol distributed under the terms of the **Creative Commons Attribution License** (https://creativecommons.org/licenses/by/4.0/), which permits unrestricted use, distribution, and reproduction in any medium, provided the original author and source are credited

5 Add an equal volume of chloroform: isoamyl alcohol (24:1, v/v) (600 µL) and mix by inverting 10-15 times. Ensure the organic and aqueous phases become mixed at least temporarily.

6 Separate the phases by centrifuging at 16,000 rcf for 1 min at 20°C.

7 Transfer the upper aqueous phase to a new 1.5 mL Eppendorf tube.

8 Repeat the chloroform: isoamyl alcohol clean (equal volume).

DNA PRECIPITATION

9 Add 1.5x volume of 100% ethanol (~900 µL) and 0.1x volume of 3 M sodium acetate pH 5.2 (~60 µL). Incubate on ice for 1 min.

Sample volume should be a maximum 600 µL after cleaning with chloroform: isoamyl alcohol.

10 Centrifuge at 16,000 rcf for 1 min. Carefully decant the supernatant as soon as possible, without disturbing the pellet.

High-molecular weight DNA should pellet easily, thus could pulse spin for 10 s at maximum speed. If no pellet can be seen, centrifuge for longer. Bringing ethanol concentration to 2x volume is also an option.

11 Add approximately 700 µL of freshly prepared 70% ethanol, enough to cover the pellet. Let the pellet soak for 1 min at room temperature to dissolve excess salts.

12 Centrifuge at 16,000 rcf for 1 min at 4°C. Carefully decant the supernatant as soon as possible, without disturbing the pellet.

13 Repeat previous steps for a second 70% ethanol wash.

14 Air-dry the pellet by placing the tube upside-down on tissue paper for 5-15 min (until all ethanol has evaporated). Be careful of water touching or dislodging the pellet.

15 Dissolve DNA with 60 µL of 10 mM Tris-HCl pH 8.

Citation: Ashley Jones, Neeraj Purushotham, Jamila Nasim, Benjamin Schwessinger (04/09/2020). DNA clean-up and size selection for long-read sequencing. https://dx.doi.org/10.17504/protocols.io.betdjei6

This is an open access protocol distributed under the terms of the **Creative Commons Attribution License** (https://creativecommons.org/licenses/by/4.0/), which permits unrestricted use, distribution, and reproduction in any medium, provided the original author and source are credited

16 Quantify the DNA on a Nanodrop and a Qubit fluorometer (dsDNA broad-range assay).

NEEDLE SHEARING (OPTIONAL)

17 Needle shearing is optional and helps provide a more uniform DNA length. This can maximise long-read sequencing output at the expense length. For instance, repetitive polyploid plant DNA over 25 kb can block nanopores, stopping sequencing prematurely.

Choose a needle and a syringe; increasing needle gauge (smaller diameter), causes more DNA shearing.

Needle	Passes	Estimated N50
29 gauge	5	10-20 kb
26 gauge	5	20-35 kb

- *No difference was found between 5, 10, 15 and 20 passes using the 29 gauge needle with plant DNA. The 26 gauge needle has not been extensivley tested.*
- *The chloroform clean-up and DNA precipitation from previous sections has already caused some DNA shearing.*

18 Aliquot 3-9 µg of DNA into a 1.5 mL Eppendorf DNA LoBind tube. Adjust the volume to exactly 60 µL using 10 mM Tris-HCl (pH 8).

19 Connect the needle to a syringe and perform 5 passes with DNA (aspirate and dispense 5 times). Proceed directly to short-read elimination.

SHORT-READ ELIMINATION

20 Choose a Short-Read Eliminator (SRE) product supplied by Circulomics. Recovery estimated based on high-molecular weight DNA from non-model species. Expect higher loss if DNA is significantly sheared.

Product	Catalogue number	Size selection	Advertised recovery	Realistic recovery
SRE XS	SKU SS-100-121-01	≥ 10 kb	50-90%	50-75%
SRE	SKU SS-100-101-01	≥ 25 kb	50-70%	30-60%
SRE XL	SKU SS-100-111-01	≥ 40 kb	40-50%	< 45%

21 Aliquot 3-9 µg of DNA into a 1.5 mL Eppendorf DNA LoBind tube. Adjust the volume to exactly 60 µL using 10 mM Tris-HCl (pH 8) or Buffer EB (supplied by Circulomics).

DNA mass must be measured by Qubit Fluorometer (Thermo Fisher Scientific) or equivalent

Citation: Ashley Jones, Neeraj Purushotham, Jamila Nasim, Benjamin Schwessinger (04/09/2020). DNA clean-up and size selection for long-read sequencing. https://dx.doi.org/10.17504/protocols.io.betdjei6

This is an open access protocol distributed under the terms of the **Creative Commons Attribution License** (https://creativecommons.org/licenses/by/4.0/), which permits unrestricted use, distribution, and reproduction in any medium, provided the original author and source are credited

- *DNA mass must be measured by Qubit Fluorometer (Thermo Fisher Scientific) or equivalent.*
- *DNA must not contain high levels of salts, polyphenols, polysaccharides or other contaminants.*

22 Add 60 µL of Buffer SRE to the sample (supplied by Circulomics). Mix thoroughly by gently tapping the tube or by gently pipetting up and down.

23 Centrifuge at 10,000 rcf for 30 min at room temperature. Note where the DNA will pellet, by marking the tube or placing the tubes in a consistent orientation within the rotor.

Recovery will be impacted if centrifugation is performed at low temperature (e.g. 4°C).

24 Carefully remove the supernatant with a pipette as soon as possible, without disturbing the pellet. Care must be taken as the pellet is fragile, being easily dislodged.

The DNA pellet may not be visible. Be careful of accidentally losing the pellet; mark the expected spot.

25 Add 200 µL of freshly prepared 70% ethanol, enough to cover the pellet. Do not tap or mix after adding ethanol.

26 Centrifuge at 10,000 rcf for 2 min at room temperature. Carefully remove the supernatant with a pipette as soon as possible, without disturbing the pellet.

27 Repeat previous steps for a second 70% ethanol wash.

28 Air-dry the pellet by placing the tube upside-down on tissue paper for 5-15 min (until all ethanol has evaporated). Be careful of water touching or dislodging the pellet.

29 Add 50 µL of Buffer EB (supplied by Circulomics). Mix by gently tapping the tube. Pellet should hydrate at room temperature within 10 min. If not, incubate at 50°C for 10 min.

Original Circulomics protocol recommends 50°C for 1 h (excessive). DNA concentration increased < 8%. Limit DNA shearing by avoiding high temperatures. Pellet will hydrate further when stored at 4°C.

30 After incubation, gently tap the tube to ensure that the DNA is properly resuspended and mixed.

Citation: Ashley Jones, Neeraj Purushotham, Jamila Nasim, Benjamin Schwessinger (04/09/2020). DNA clean-up and size selection for long-read sequencing. https://dx.doi.org/10.17504/protocols.io.betdjei6

This is an open access protocol distributed under the terms of the **Creative Commons Attribution License** (https://creativecommons.org/licenses/by/4.0/), which permits unrestricted use, distribution, and reproduction in any medium, provided the original author and source are credited

31 Quantify the DNA on a Nanodrop and a Qubit fluorometer (dsDNA broad-range assay).

32 Store DNA at 4°C to prevent cycles of freeze-thawing that shear the DNA.

No effects on DNA integrity have been noticed for samples stored at 4°C for extended periods.

33 EXPECTED RESULTS

Fresh mycelia (approximately 2 g) was used to extract crude high-molecular weight DNA using a CTAB method previously described. See Jones *et al.* (2019) High-molecular weight DNA extraction from challenging fungi using CTAB and gel purification, Protocols.io. The DNA was cleaned and then size selected with a Short-Read Eliminator kit (SRE), as described in this protocol. Initially the Qubit:Nanodrop ratio had a large discrepancy (Table 1 and Figure 1). This is due to the presence of RNA, given the 260/280 is above 1.8. Cleaning removed this discrepancy, particularly with the removal of all RNA. With RNA not present, the 260/230 ratio is more representative, despite appearing worse.
For sequencing, we used a MinION sequencer from Oxford Nanopore Technologies, creating a native genomic DNA library (SQK-LSK109) following the manufacturer's instructions. Although the DNA was not perfect, a high sequencing output was achieved (Table 2). Size selection with SRE resulted in a long-reads and a high N50 (Figure 2).

Citation: Ashley Jones, Neeraj Purushotham, Jamila Nasim, Benjamin Schwessinger (04/09/2020). DNA clean-up and size selection for long-read sequencing. https://dx.doi.org/10.17504/protocols.io.betdjei6

This is an open access protocol distributed under the terms of the **Creative Commons Attribution License** (https://creativecommons.org/licenses/by/4.0/), which permits unrestricted use, distribution, and reproduction in any medium, provided the original author and source are credited

Table 1: Quantification of the crude DNA, DNA after cleaning and after size selection.

Sample	DNA input µg	SRE kit	Qubit ng/µL	Nano ng/µL	Qubit: Nano	260/ 280	260/ 230	Vol µL	Yield µg (Qubit)
Mp strain 1 crude DNA	NA	NA	64.30	526.80	1:8.19	2.05	2.09	300	19.29
Mp strain 1 clean DNA	19.00	NA	271	431.80	1:1.59	1.92	2.16	50	13.55
Mp strain 1 clean + SRE	9.00	25 kb	74.10	97.60	1:1.31	1.88	1.67	50	3.71
Mp strain 2 crude DNA	NA	NA	258	3,025	1:11.72	2.06	2.06	300	77.40
Mp strain 2 clean DNA	19.00	NA	272	280.10	1:1.03	1.80	1.35	50	13.60
Mp strain 2 clean + SRE	9.00	25 kb	94	146.10	1:1.55	1.82	1.29	50	4.70

(A) *M. phaseolina* before clean

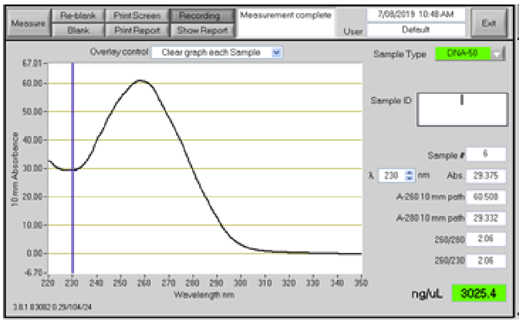

(B) *M. phaseolina* after chloroform cleaning

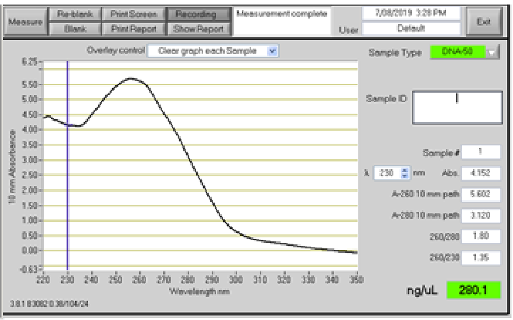

(B) *M. phaseolina* after clean and short read eliminator

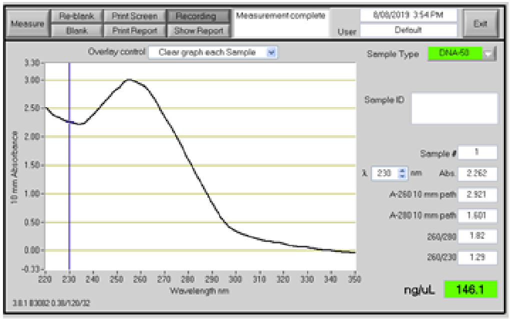

Figure 1: Spectrophotometer results of DNA at multiple stages of the protocol. Strain 2 is shown as an example. (A) Initial DNA before cleaning. (B) DNA after an initial clean with chloroform and ethanol precipitation. (C) DNA after cleaning with chloroform and size selecting with a short read eliminator kit (Circulomics). Readings taken using 1 µL on a Thermo Scientific Nanodrop 1000.

Citation: Ashley Jones, Neeraj Purushotham, Jamila Nasim, Benjamin Schwessinger (04/09/2020). DNA clean-up and size selection for long-read sequencing. https://dx.doi.org/10.17504/protocols.io.betdjei6

This is an open access protocol distributed under the terms of the **Creative Commons Attribution License** (https://creativecommons.org/licenses/by/4.0/), which permits unrestricted use, distribution, and reproduction in any medium, provided the original author and source are credited

Table 2: Sequencing results with a single MinION flow cell per sample (FLO-MIN 106 R9.4.1 revD).

Sample	SRE ≥	Library input µg	Loaded µg	Library preparation	Pores	Output Gb	Base call Gb	N50 kb
Mp strain 1	25 kb	3.00	1.00	Ligation SQK-LSK109	1,557	13.71	13.29	21.75
Mp strain 2	25 kb	3.00	0.94	Ligation SQK-LSK109	1,362	9.72	9.57	43.50

(A) Sorghum rot *Macrophomina phaseolina* strain 1; short read eliminator 25 kb, 13.71 Gb, N50 21.75 kb.

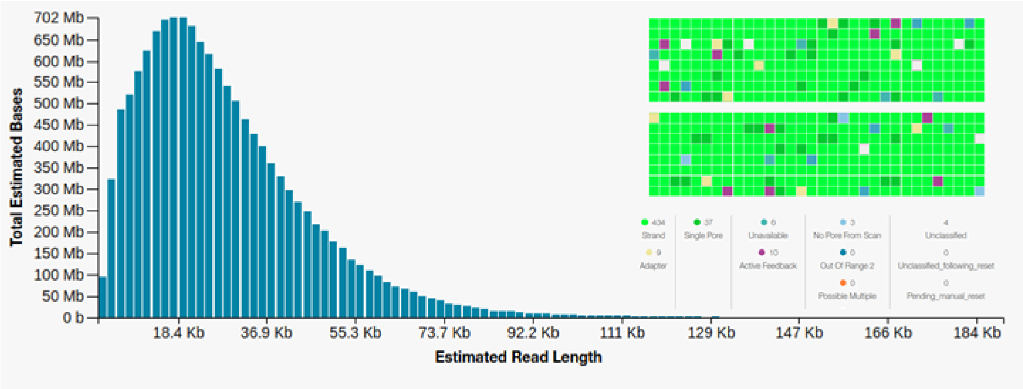

(B) Sorghum rot *Macrophomina phaseolina* strain 2; short read eliminator 25 kb, 9.72 Gb, N50 43.50 kb.

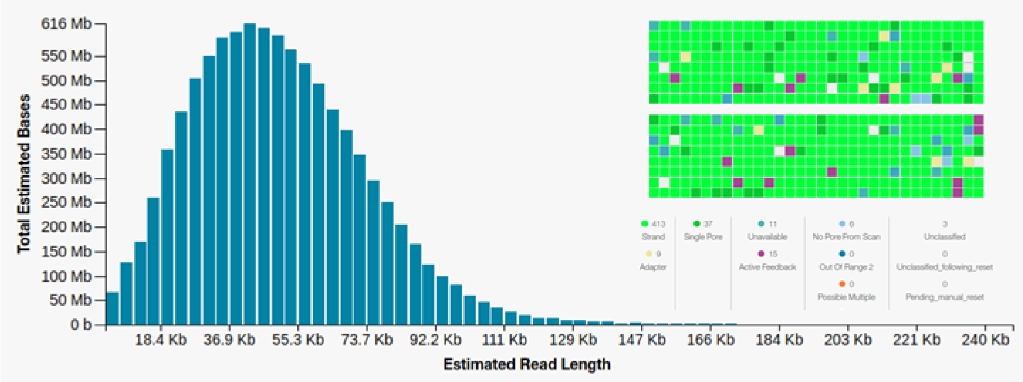

Figure 2: Expected read length histograms on MinKNOW. Both sorghum rot *Macrophomina phaseolina* strains were size selected with a short read eliminator kit (Circulomics) for 25 kb and above. DNA libraries were then prepared with an end ligation kit (Oxford Nanopore SQK-LSK109). Inserts show pore usage, light green indicates pore is active and sequencing is occurring.

Citation: Ashley Jones, Neeraj Purushotham, Jamila Nasim, Benjamin Schwessinger (04/09/2020). DNA clean-up and size selection for long-read sequencing. https://dx.doi.org/10.17504/protocols.io.betdjei6

This is an open access protocol distributed under the terms of the **Creative Commons Attribution License** (https://creativecommons.org/licenses/by/4.0/), which permits unrestricted use, distribution, and reproduction in any medium, provided the original author and source are credited
