# Supplementary material for: High-molecular weight DNA extraction, clean-up and size selection for long-read sequencing
Source: PLoS One. 2021 Jul 15;16(7):e0253830. doi: 10.1371/journal.pone.0253830 (PMC8282028; doi:10.1371/journal.pone.0253830)
Supplement: Attachment — Submitted filename: response_to_reviewersBS.docx [file pone.0253830.s002.docx]

**Response to reviewers**

Dear reviewer,

Thank you for your time to provide comments and a rigorous review of the manuscript, the exchange has been helpful and insightful. Please find our responses to each point below, which have been incorporated into a revised, improved manuscript.

**1-5)** Author and reviewer are in agreement PLOS ONE standards have been fulfilled. No further action required.

**6)** **“*Line 26: the authors claim that the protocol works for both ONT and PacBio sequencing whereas they only present the data for ONT.”***

An example is now provided for a plant PacBio Sequel II HiFi dataset in Table 2 (*Wahlenbergia ceracea*). DNA quality and length generated from the protocol meets the submission requirements for common sequencing centres and platforms (ONT, PacBio, Illumina etc).

***“Line 30: “eucalypts” is wrongly spelled.”***

For simplicity and clarity, this has since been removed.

***“Line 30: The authors claim that this DNA isolation protocol has been used for several organisms such as eucalyptus, acacias, rice, themeda, wheat, various fungi, geckos, skinks, ticks, ladybird beetles, caterpillars and E. coli but there is no literature reference pointing to the publications where this protocol was used for the above-mentioned species.”***

A more extensive Table 2 is now provided, which includes examples from plants, fungi, bacteria and animal. Many other species have been done, some in workshops and teaching courses however will not be presented here. For simplicity and clarity, parts of this text in the manuscript have now been removed.

***“Line 84: “includes quality reads over”. Please mention the q-score for referring a read as high quality.”***

Q scores (Phred scale) have now been mentioned in text, mean and median read qualities have been added to Table 2.

***“Table 1: The authors should mention if 10 gm was fresh wait or powdered tissue.”***

Added to Table 1 (fresh).

***“Table 2: I would also like to see the mean and the median read length together with N50 in this table. Also, the authors should state the Guppy (or Bonito) version used for base-calling.”***

The statistics mean read length, median read length (amongst others including mean read quality, median read quality), have been added to a more extensive Table 2. The ONT basecaller (Guppy version 4.4.1) has now been added to the caption of Table 2

**7)** NA. No further action required.

Kind regards,

Ashley Jones
